# **Condition monitoring and fault diagnosis of power transformer based on non-invasive measurement**

**DOI:** 10.1038/s41598-025-14242-2

**Published:** 2025-09-01

**Authors:** Mohammed Youssef, El-Said Abdelaziz, Hassan S. Mohamed, Mohammed Attia

**Affiliations:** 1 Electrical Power and Machines Department, Higher Institute of Engineering, El Shorouk Academy, Cairo, Egypt; 2 Department of Electrical Engineering, Faculty of Engineering, Al Azhar University, Cairo, Egypt

**Keywords:** Power transformer monitoring, Abnormal condition *-* internal faults, Artificial neural network (ANN), Lear vector quantization (LVQ), Electrical and electronic engineering, Software

## Abstract

In modern power systems, it is crucial to monitor and detect internal faults in power transformers promptly and accurately to ensure reliability and prevent disruptions. Failure to identify these faults promptly can reduce the transformer’s lifespan, cause system disconnection, and compromise network stability. This paper introduces an innovative method for the discrimination, classification, and localization of internal short-circuit faults in power transformers, with a focus on three types of winding faults: turn-to-turn fault, series short circuits, and shunt short circuits. The proposed method introduces an online detection scheme utilizing the ΔV-I_in_ locus diagram, which leverages existing measurement devices without requiring additional hardware. A comprehensive winding model was developed in MATLAB to simulate insulation failures, and the method also analyzes the effects of faults and harmonic distortions on transformer performance. Features for fault discrimination and localization are derived from the ΔV-I_in_ locus and calculated using the practical design specifications of three power transformer models with capacities of 3 MVA, 5 MVA, and 7 MVA, operating at 50 Hz in a three-phase configuration. Experimental results on the 3 MVA transformer demonstrate that the formulated identifier efficiently detected all three types of insulation breakdown with an accuracy of 98.51%. Additionally, the fault localization algorithm achieved a fault location accuracy of approximately 93.28%. The findings indicate that the proposed approach is a robust and reliable tool for assessing the condition of power transformers.

## Introduction

Transformers act as a significant function in power systems by providing a main connection between the production and consumption of electricity. Ensuring the reliability of power transformers, particularly by preventing faults that could lead to transformer failure, is essential for maintaining network stability. In recent years, online transformer monitoring has garnered widespread recognition for its efficacy in rapidly detecting faults, prevent complete transformer shutdowns, enhance system reliability, and provide superior service to consumers ^1^.

Traditional differential relays, despite utilizing terminal current waveforms, lack the sensitivity to detect minor internal faults, with their performance and settings heavily dependent on specific operational parameters ^1,2^. To address these limitations, recent advancements in transformer protection have introduced methods aimed at enhancing fault detection accuracy and sensitivity. These techniques are broadly categorized into three groups:

1. Methods based on Current/Voltage.
2. Methods based on Frequency.
3. Methods based on Flux.

### Methods based on current/voltage

Current/voltage‐based methods use terminal parameters of a transformer for internal faults protection. Researchers widely study such methods. These methods exhibit general shortcomings, including instrument saturation and associated errors. The smallest internal fault minimally affects the terminal parameters of the transformer, generating negative and zero sequence components of current and voltage. Methods based on positive sequence (PS), such as differential relays, exhibit lower sensitivity when compared to those based on negative sequence (NS) and zero sequence (ZS). A healthy transformer consists solely of the primary side (PS) component, while the secondary side (NS) component is deemed insignificant. The asymmetrical components in currents and voltages are attributed to internal faults in transformers.

The NS-based methods in references ^3–5^ compute the NS line currents during the preliminary phases of the detection technique. The discrete Fourier transformation is employed to estimate the magnitude and phase angle of the NS. Differential NS current can differentiate between internal and external faults. Previous studies ^3–5^ indicate that the sensitivity of the NS method (3%) surpasses that of the differential relay (10%).

The primary drawback of the NS current-based method is the potential for mal-operation caused by differential current during the energization process. The NS voltage serves as a detection mechanism for inrush conditions, as indicated in ^6^. This is due to the fact that the voltage on both sides of the transformer remains unaffected during energization by the inrush current. In ^7,8^, the authors have introduced a comparison for the ratio of both the primary and secondary NS line currents ($RNSC$) and the transformer’s turns ratio as shown below:

1 $${\text{Detector}} = RNSC - \frac{{N_{2} }}{{N_{1} }}$$

Recently, in order to solve the problems of NS methods, A method based on symmetrical components has been introduced in ^9^. This method identifies a useful adaptive characteristic plot for NS relays. The method employs the vector difference between the positive sequence impedance $(\Delta {\overline{Z} }_{\text{Pos}})$ and the negative sequence current component $\left(\Delta {\overline{I} }_{\text{Neg}}\right)$ to detect internal faults.

A Fourier-based analytical method is introduced in ^10^, demonstrating efficacy in detecting internal faults under light load conditions. This approach necessitates adherence to IEEE standards for harmonic distortion limits in the input voltage. Complementing this^11^, proposes a coefficient calculation algorithm derived from primary current and voltage measurements. The algorithm establishes baseline coefficients under healthy operating conditions by analyzing neutral current patterns, enabling real-time detection of Turn-Turn Faults (TTF). However, its implementation requires prior characterization of the load profile, limiting applicability in dynamic grid environments.

To identify diverse electrical and mechanical faults in transformers, an online monitoring technique was proposed in ^12^. This method employs a diagnostic approach by analyzing the input current locus diagram plotted against the differential voltage (between input and output voltages), which is then compared to reference data from a healthy condition. Faults are indicated by deviations in the shape of the elliptical locus, which is generated for faults types including axial displacement, turn-turn faults, buckling stress, disk space variation, and varying power factors. While each fault alters the locus, pinpointing the root cause based solely on geometric changes is challenging. Minor internal faults have negligible effects on the locus diagram, whereas severe faults cause significant shifts in its area and rotational orientation. The ΔV-I locus method can detect faults with severity as low as 5%, though it requires baseline data from healthy conditions for comparative analysis. A key limitation, however, is the difficulty in distinguishing between fault types solely based on changes to the locus’s rotation and area parameters.

The Extended Park’s Vector Technique (EPVT) is an online diagnostic technique that employs dual-winding current waveform analysis for internal fault detection in transformers ^13–16^. Characterized by its robustness, simplicity, and efficacy, The methodology employs frequency-domain spectral analysis of the alternating current component within the Park’s vector modulus, extracted from the transformer’s on-load excitation currents. In recent years, EPVA has been increasingly applied to fault diagnosis in electrical systems, including turn-to-turn fault (TTF) detection in power transformers and stator winding fault identification in both synchronous and induction motors. The methodology entails calculating differential currents for each phase, followed by EPVA-based derivation of the corresponding d-q axis components from these differential currents.

Even though the sensitivities of instrument transformers (CT and VT) are reduced to a certain extent, these methods are still non-invasive. Table 1 presents a comparative analysis encompassing the key strengths, limitations of the evaluated methodologies.

**Table 1:** Comparative analysis of current/voltage-based techniques for internal fault discrimination.

Method	References	General features	Non‐invasive/ invasive	Sensorless/ Sensor	Online	Offline
Differential relay	^1,2^	Low sensitivity	Non	Sensorless	✓	✓
		Very simple and robust				
		Mal-operation occurrence during operation				
		Effect of CT saturation, magnetizing current and errors				
Negative sequence	^6–9^	Reasonable sensitivity (3%)	Non	Sensorless	✓	__
		Production of negative current in case of un-grounded faults				
		Impact of CT saturation, magnetizing current and errors				
		voltage transformer limitations				
		each side of the transformer must be loaded				
		Algorithms do not consider incipient nature				
Negative Sequence-Based Positive Impedance Method	^9^	Error in case of online computations	Non	Sensorless	✓	__
		Simple calculation process				
		Reasonable sensitivity (2.5%)				
		OLTC operation adaptation				
		Still stable at 10% over-excitation				
		In case of unbalanced load and source, the algorithm still stable				
		localization the internal fault cannot be achieved				
Zero sequence	^22,23^	Errors due to using CT and VT	Non	Sensorless	✓	__
		Still stable in inrush current cases				
		Needs calibration in delta winding connection				
		The healthy condition data must be existed				
		Valid for transformer bank only				
ΔV‐I locus diagram	^12^	Can deal with various faults	Non	Sensorless	✓	__
		Errors due to using CT and VT				
		The healthy condition data must be existed				
Extended Park’s vector approach	^13–16^	Cannot acts well for unbalance load	Non	Sensorless	✓	__
		Cannot operate in transformer energization				
		Measuring instruments error				
		Can detect only 4% turns faults				
Magnetic Flux Test	^24^	Can detect the faulty phase	Non	Sensorless	__	✓
		The implementation for Y-connection for 3-phase transformer				
		Cannot applied for five-leg core				
		Error due to flux interception				

### Methods based on frequency

Frequency response analysis is a well‐known method for detecting various faults. This technique uses the transformer RLC network parameters variation to detect internal faults. This method’s initial application is stated in ^17^. At a later stage, the FRA response can be used as a reference for detecting fault. The behavior of this curve is influenced by the distributed elements, which encompass the capacitances present between the various turns of a winding, the turns of different windings, and the self- and mutual-inductance of the windings.

Two methods are used to generate the required frequency spectrum: injecting an impulse into the winding or employing a frequency sweep with a sinusoidal signal ^18^. The primary advantage of the impulse response approach, in contrast to other techniques, is its reduced measurement duration. Nevertheless, the frequency sweep technique, in contrast to the impulse response method, has the following advantages:

- An accepted signal-to-noise ratio,
- Acts very sensitive in all ranges,
- The technique can operate with low number of measuring instruments.

Prior studies ^19–21^ have demonstrated the efficacy of statistical parameter analysis in Frequency Response Analysis (FRA) for detecting transformer faults. This approach replaces labor-intensive graphical comparisons and expert-dependent assessments by synthesizing magnitude and phase-angle data into a unified polar plot. Quantitative evaluation of this plot is conducted using three metrics: city-block distance, root-mean-square deviation, and image-based Euclidean distance. The framework enables automated identification of critical faults, including internal winding defects, axial displacement, and turn-to-turn spacing irregularities, thereby enhancing diagnostic objectivity and operational efficiency.

Sweep Frequency Response Analysis (SFRA) facilitates the comparative assessment of multiple SFRA signatures to identify electromechanical anomalies. Frequency-dependent deviations such as resonance shifts, emergence of new resonant peaks, and spectral amplitude variations serve as robust indicators of electrical or mechanical faults. Core-specific defects are detectable within the low-frequency spectrum, as demonstrated in ^27^.

Frequency Response Analysis (FRA) is categorized as an offline method, posing significant challenges due to strict operational constraints, as utilities often cannot guarantee adequate outage availability. Although FRA can precisely identify fault types after a transformer outage, it lacks suitability for real-time monitoring. To resolve these limitations, researchers have introduced online FRA solutions; however, these methods remain unstandardized, less accurate than offline approaches, and require enhancements across multiple parameters. Recent advancements propose online FRA techniques for detecting electrical and mechanical faults in power transformers. Transitioning to an online framework, however, faces key hurdles, including ^25^:

- Signal injection system shortage,
- Difficulty isolating the transformer’s response from the operational grid,
- Need to account for the transformer’s inherent internal state variations.

Utilizing the bushing tap for signal injection in online monitoring addresses the aforementioned challenges, as directly connecting signals to high-voltage buses is impractical. However, older transformers often lack this feature ^26^. For transformers without bushing taps, a non-invasive capacitive sensor mounted on the bushing surface can provide real-time monitoring without intrusive hardware. A multi-frequency signal is applied to the transformer, and its response is measured via a Rogowski coil ^27^. By analyzing input and output signals, the transfer function can be derived during online operation ^28^.

In ^26^, researchers emphasize that this method relies on baseline healthy data to establish the FRA reference for fault detection. A key challenge in online FRA is the impact of OLTC operations, which can distort the FRA curve at low frequencies studies show this effect occurs within a range of up to 100 kHz.

Abu-Siada et al. ^29^ proposed a novel 3D frequency response analysis (3D-SFRA) technique that integrates magnitude, phase, and frequency data into a unified diagnostic signature, introducing the use of digital image processing (DIP) methods such as HOG, LBP and SIFT-3D to automate the detection of subtle mechanical faults in power transformers. The main novelty lies in transforming complex SFRA data into a 3D visual format that enables fault classification without manual interpretation. However, its limitations include reliance on baseline healthy data, often unavailable in aged transformers, transformer-specific threshold calibration, and computational complexity, which restrict its scalability and general use. In contrast, the more recent study introduced in ^30^ presents fault detection technique using 3D FRA signatures paired with CNNs and volumetric image processing, enabling deeper feature extraction and higher sensitivity to subtle anomalies. Its novelty lies in reducing dependence on reference data and improving generalization across different transformer types and fault conditions. Nonetheless, ^30^ may still face challenges related to implementation complexity and the need for validation across larger datasets. Table 2 provides a concise overview of the strengths and limitations of frequency-based diagnostic methods.

**Table 2:** FRA features for various faults approach in power transformers.

Method	References	General features	Non‐invasive/invasive	Sensor less/ Sensor	Online	Offline
Frequency response analysis	^21–27^	The ability to deal with electrical and mechanical defects in various allocation inside the transformer	Non	Both	✓	✓
		Healthy condition data must be existed				
		In traditional signature healthy condition data must be exist				
		Additional instruments may be installed				
		Localization process for the internal fault cannot acts well				
		Mal-operation occurrence				
		Detailed analysis required to improve detection accuracy				

### Methods based on flux

Magnetic flux measurement offers an alternative to terminal current and voltage analysis for detecting internal faults in power transformers. This method necessitates specialized sensors to monitor flux distribution. Finite element method (FEM) simulations of a transformer model enable comparative analysis of flux behavior under healthy and faulty conditions. For example, during an internal fault in the first section of phase V, leakage flux reroutes through the transformer tank, insulating oil, and neighboring windings, altering its path ^31^.

A search coil, consisting of a wound copper conductor, is a widely utilized sensor for magnetic flux measurement in power systems. As magnetic flux through the coil fluctuates, a voltage is induced across its terminals, directly proportional to the rate of flux change. These sensors achieve exceptional sensitivity, detecting magnetic fields as low as 2 × 10^−5^ nT, with no theoretical upper detection limit. Commonly integrated into electrical equipment such as transformers, motors, and generators, search coils are especially effective for non-invasively identifying turn-to-turn Faults (TTF) in power transformer windings, enabling precise fault localization ^33–35^.

#### Leakage flux–based methods

Internal faults in transformers generate leakage flux, which can be monitored for fault descrimination. Search coils are typically installed near the high-voltage (HV) winding at the upper and lower sections of each leg ^33,34^. However, these coils require bulky insulation to meet HV clearance standards, altering transformer design and tank dimensions.

A non-invasive leakage flux–based method proposed in ^35^ addresses these limitations by deploying novel sensors near the transformer core, bypassing HV winding installation. These sensors enhance sensitivity, are retrofittable to existing transformers, and enable online/offline detection of internal faults, including precise identification of the faulty phase and location.

#### Core flux–based methods

The Core Flux-Based (CFB) method offers a straightforward, sensitive, and reliable approach for detecting internal faults in power transformers during online or offline condition monitoring, with the ability to pinpoint fault locations. This technique computes flux linkage increments using transformer equations to identify anomalies ^31^.

Alternatively, a method employing three search coils per phase, installed around the HV winding to monitor leakage flux ^36^, enables online fault detection and localization. However, its practicality is limited by insulation requirements at medium voltage (MV) levels. Positioning the sensors away from the core reduces sensitivity, as core flux induces uniform voltages across sensors under normal operation. During internal faults, these voltages diminish, allowing fault localization by comparing voltage discrepancies between sensors within each phase.

#### Transformation action–based method

Recent advancements utilizing search coils ^39^ have expanded their application to detect both coil displacement and Turn-to-Turn Faults (TTF). While current configurations achieve a sensitivity of 2.5%, limited by fewer coil turns, this can be enhanced by increasing the number of turns ^37^. The method demonstrates robustness under diverse operating conditions, including inrush currents, over-excitation, current transformer (CT) saturation during faults, and On-Load Tap Changer (OLTC) operations. However, its use is currently restricted to medium-voltage (MV) applications due to performance degradation under extra-high voltage conditions. Fault detection relies on an algorithm that calculates the voltage ratio between adjacent search coils to identify defects.

Flux-based methods, which employ sensors like search coils, offer high accuracy and sensitivity for internal fault detection. While these techniques outperform other diagnostic approaches, they are typically invasive unless coils are integrated during transformer fabrication. Retrofit installations during maintenance or initial construction render them non-invasive. Table 3 summarizes these characteristics, highlighting trade-offs between sensitivity, invasiveness, and practical implementation constraints.

**Table 3:** Comparison of flux-based methods for internal fault detection and localization in power transformers.

Method	References	General features	Non‐invasive/ invasive	Sensorless/ Sensor	Online	Offline
Leakage flux	^33,34^	Can deal with winding movement	Non	Sensor	✓	✓
		Can detect the faulty phase				
		Insulation process for the high voltage side in case of search coil				
		The ability for detection the intermediate faulty winding				
		Search coil must be attached				
Core flux	^32,38,39^	Very simple	Non	Sensor	✓	✓
		More accurate				
		Very high sensitivity (0.1%)				
		Can detect the faulty phase				
		Achieve the localization process				
		Proper performance in all abnormal conditions				
		Search coil must be attached				
Linkage flux	^31^	Complex equation is needed	Non	Sensorless	✓	__
		Can detect the faulty phase				
		Very stable performance in abnormal condition				
		Low sensitivity level (10%)				
Transformer action	^37^	Can detect 2.5% faulty discs	Non	Sensor	✓	__
		The ability to deal with winding movement				
		Valid for high voltage side only				
		Very stable performance in abnormal condition				

## Paper contribution

In this paper, an online strategy is developed to monitor the operating conditions of a connected transformer and evaluate internal fault conditions. The scheme leverages the relationship between ΔV and I_in_, which forms an elliptical locus whose dimensions vary based on the fault type. This characteristic allows the scheme to extract new features effectively, enabling the identification and mapping of faults within power transformer. To address internal insulation failure in transformer windings, two approaches are proposed and implemented. The first approach classifies three types of internal insulation faults using an artificial neural network with high accuracy, while the second approach determines the precise location of these faults along the transformer winding by dividing it into sections.

The determination of significant features for internal faults identification in power transformers remains a pivotal and evolving area of research. This study presents an innovative advancement by estimating and calculating five novel features derived from the ΔV-I_in_ locus. These features offer substantial improvements in the research outcomes, as outlined below:

1. The extracted features enable the Artificial Neural Network (ANN) algorithm to achieve exceptional accuracy in internal fault classification, reaching 98.51% across training, testing, and validation phases.
2. The features have been graphically represented for two different transformer models to demonstrate their capability to effectively distinguish between various fault types.
3. Additionally, these features enable precise fault localization along the transformer winding, delivering high accuracy and simplifying maintenance processes.
4. A detailed and updated methodology for deriving these five novel features is provided in Sect. “Features extraction discrimination technique” of the paper.
5. These features serve as a distinctive fingerprint for any power transformer, as they are based on equations utilizing voltage and current data.

**Fig. 1:**
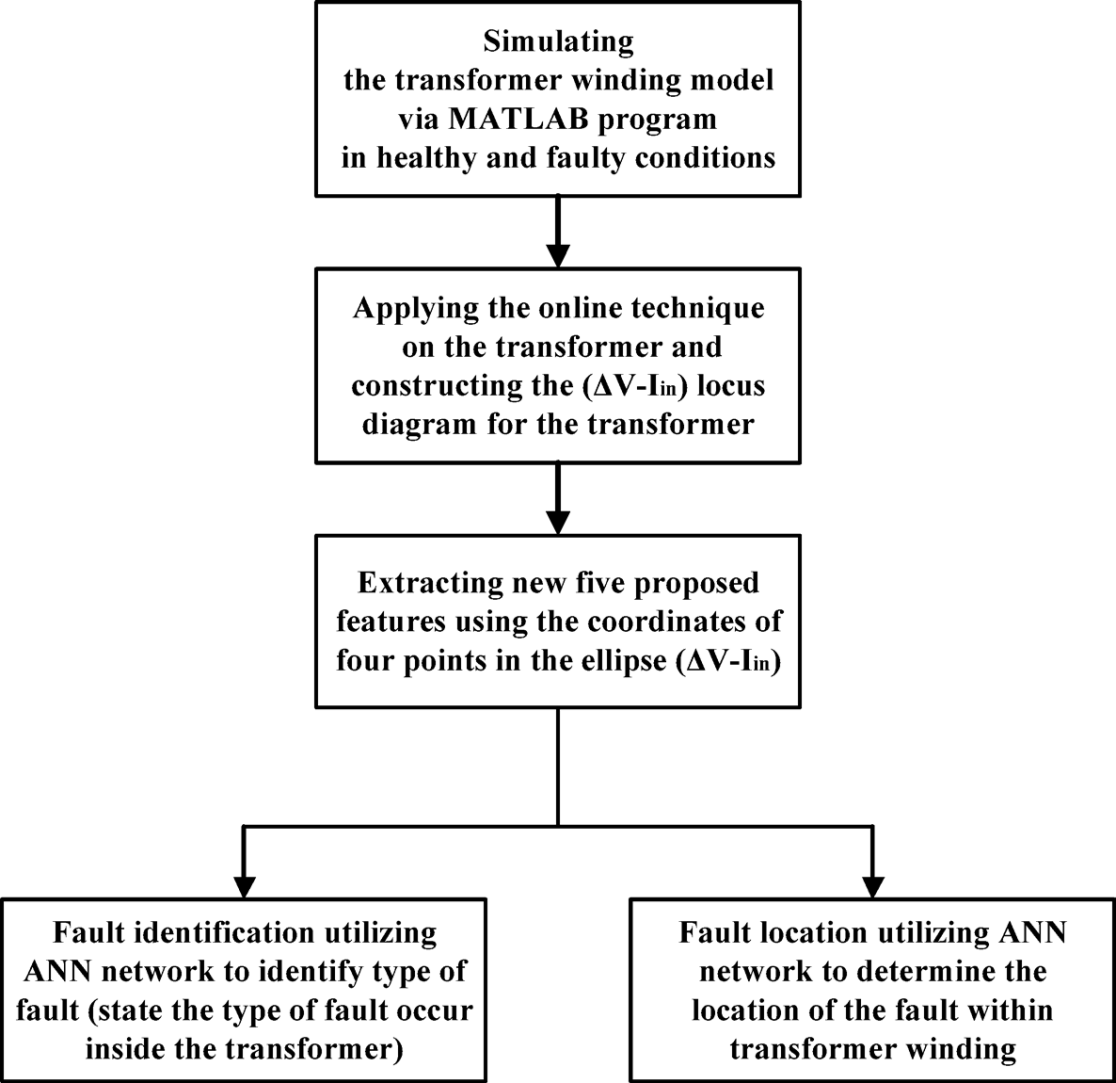
Flowchart of the proposed approach.

This contribution underscores the potential of these novel features in enhancing the reliability and efficiency of transformer fault diagnosis and maintenance. For clarification, Fig. 1 illustrates the stages applied to classify and locate the most common mechanical and electrical failures within power transformer windings.

## Transformer model contains internal faults

**Fig. 2:**
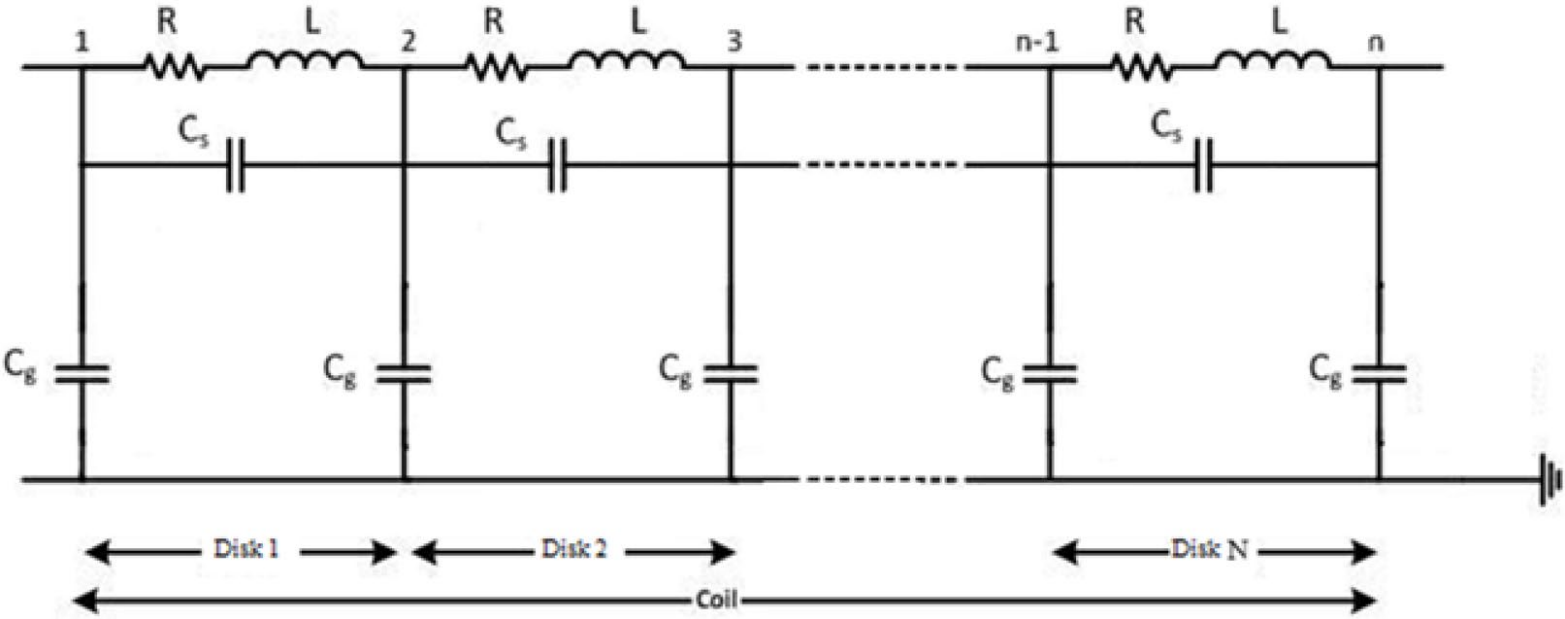
Power transformer internal winding Equivalent circuit (Disk type).

The ability to accurately predict transformer behavior during an interruption is crucial for creating effective transformer fault detection technologies. The transformer model under study depends on dividing the winding into identical sections, which enables the simulation of a number of disks along the winding. A lumped RLC circuit can simulate transformer winding very accurately and effectively. In this paper, three different transformer models with different technical specifications are simulated. The components of the transformer models (3, 5, and 7 MVA) are described in ^40^. Figure 2 shows the applied model of the winding for the power transformer.

The equivalent circuit of the transformer, depicted in Fig. 2, is employed in this study. The delta connection of the disk-type winding at the high-voltage terminal is modeled using a network with lumped parameters. Internal faults are simulated by altering the electrical parameters of the winding, such as resistance (R), series inductance (L), shunt capacitance (C_g_), and series capacitance (C_s_). The parameters of the transformer model, corresponding to various types of internal faults, are presented in Table 4.

**Table 4:** Parameters of the model and the faults that impact them.

Model parameter	Type of fault
Resistances ($R$)	Disk broking, caulking damage and wearing of tap changer
Series inductances ($L$)	Disk misrepresentation, local failure, core perversion, and winding short circuit
Series capacitances (${C}_{s}$)	Insulation aging, moisture infiltration, and disk displacement
Shunt capacitances (${C}_{g}$)	Disk displacement, large mechanical forces buckling, ingress moisture

Three different transformer models with different technical specifications are simulated. Each transformer has a different power rating with physical characteristics, such as the number of discs in the HV winding, the outer diameter of the HV winding, the inner diameters of the HV winding, and the number of turns. These physical characteristics directly affect the RLC electrical parameter models. Table 5 introduces detailed parameters of 3, 5, and 7 MVA power transformers, showing the data for each transformer ^40^.

**Table 5:** Detailed parameters of 3, 5 and 7 MVA power transformers.

Model			Transformer 1	Transformer 2	Transformer 3
1	$P$	Power rating	3 MVA	5 MVA	7 MVA
2	$V$	Voltage rating	33/11 kV	33/11 kV	20/6 kV
3	$D$	Number of discs on the HV winding	89	67	69
4	$OD$	Outer diameters of the HV winding	582 mm	609 mm	702 mm
5	$ID$	Inner diameters of the HV winding	496 mm	503 mm	579 mm
6	$T$	Number of the turns in the HV winding	1428	1206	1104
7	$R$	Resistance per disc	1.4 Ω	2.4 Ω	0.43 Ω
8	$L$	Total inductance per disc	0.16 mH	0.11 mH	0.025 mH
9	${C}_{g}$	Ground capacitance per disc	0.08 pF	0.056 pF	0.12 pF
10	${C}_{s}$	Series capacitance per disc	2.1 pF	9.0 pF	5.1 pF

## Proposed method

**Fig. 3:**
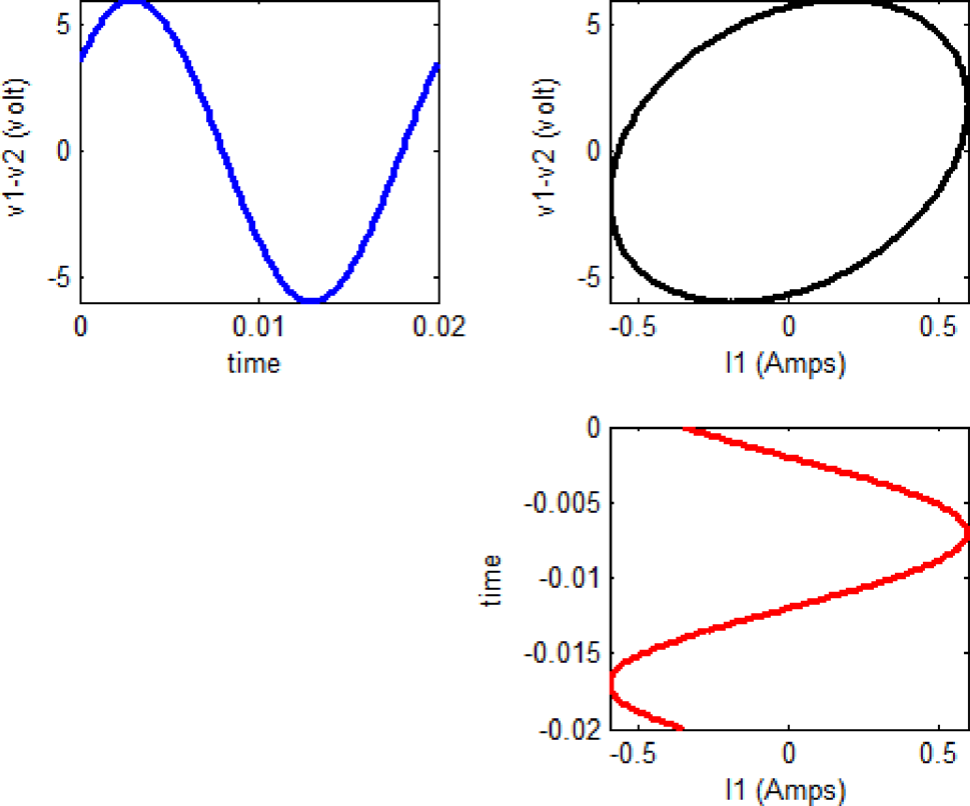
Graphical illustration of ΔV–Iin relationship.

The main objective of any diagnostic approach is to identify physical breakdown in a transformer resulting from internal faults by leveraging its sensitivity to variations in distributed inductances and capacitances ^12^. This paper focuses on the analysis of input voltage (V_in_), input current (I_in_), and output voltage (V_out_) at the rated power frequency (50 Hz) during each complete power cycle. The proposed online monitoring system uses a locus diagram to detect physical variation in the configuration of transformer winding. As shown in Fig. 3, In this diagram, the x-axis represents the input current of the transformer, while the y-axis shows the voltage difference (∆V = V_in_—V_out_) between the input–output voltages of a specific phase. This monitoring method, first introduced in ^38^, was developed to identify mechanical faults in single-phase transformers. The relationship between ∆V and I_in_ typically forms an elliptical pattern, as illustrated in Fig. 3. It is projected that each type of fault will generate a distinct (∆V–I_in_) locus, which can be used to identify and differentiate faults through the proposed monitoring scheme.

**Fig. 4:**
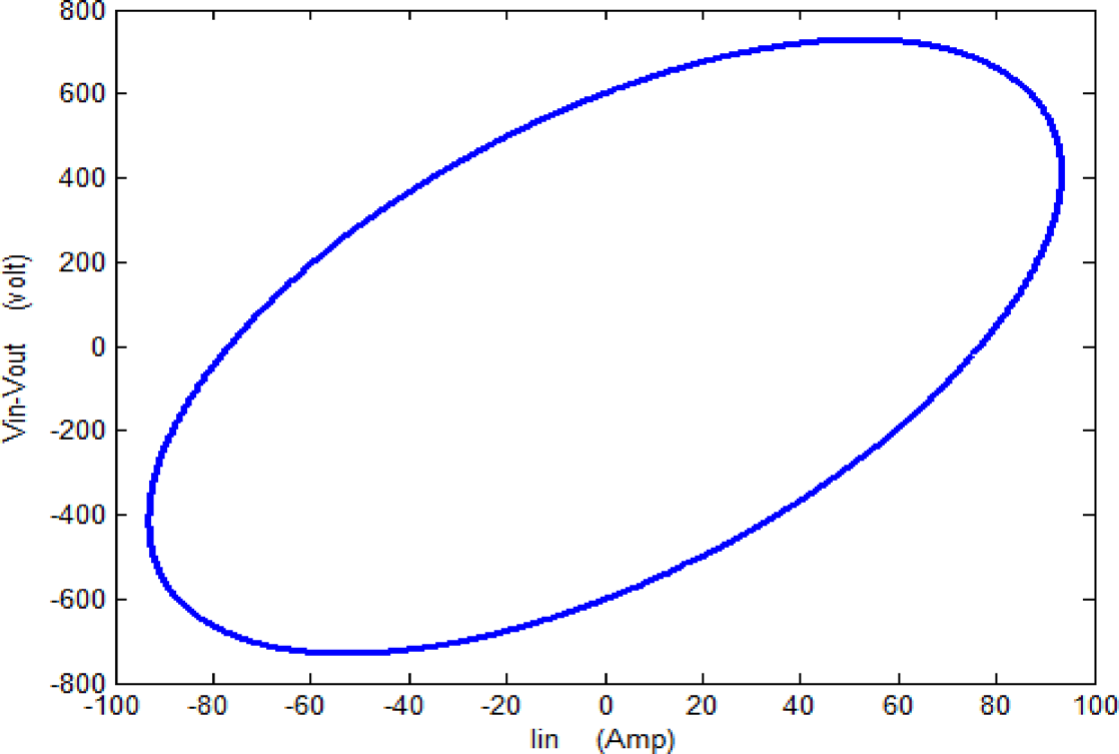
ΔV–Iin locus for a 15-kVA, 2300/230-V single-phase transformer.

The proposed approach tested by a 15 KVA, 2300/230 V single phase transformer with the following equivalent circuit parameters referred to the low voltage side as: $R_{{{\text{eq}}}} = 4.45 \Omega ,X_{{{\text{eq}}}} = 6.45 \Omega ,X_{m} = 11 K\Omega ,R_{c} = 105 K\Omega$. the (ΔV–I_in_) locus is displayed as shown in Fig. 4, noted that the transformer operates at 0.8 lagging power factor.

**Fig. 5:**
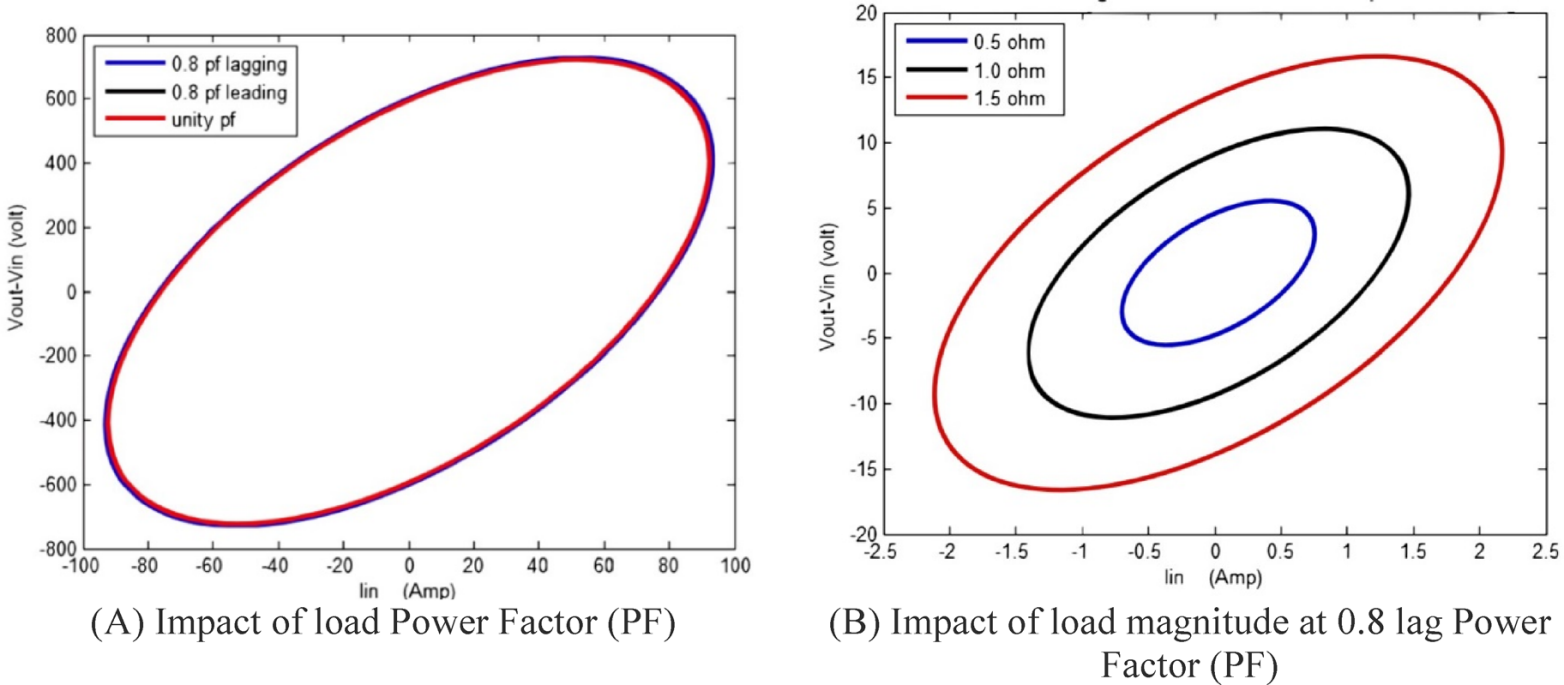
Impact of load Power Factor (PF) and load magnitude on a 15 KVA, 2300/230 V single phase transformer.

To analyze the impact of load power factor and magnitude variations on the proposed locus, simulations were conducted for a 15 kVA, 2300/230 V single-phase transformer (Fig. 5). Three operating conditions: 0.8 lagging, 0.8 leading, and unity power factor, are examined with a constant impedance magnitude, and the corresponding loci for each case are constructed, as shown in Fig. 5A.

Similarly, to investigate the effect of load magnitude variation on the proposed locus, different load levels are simulated while maintaining a constant power factor. The results are illustrated in Fig. 5B.

## Simulation result for healthy and abnormal conditions

**Fig. 6:**
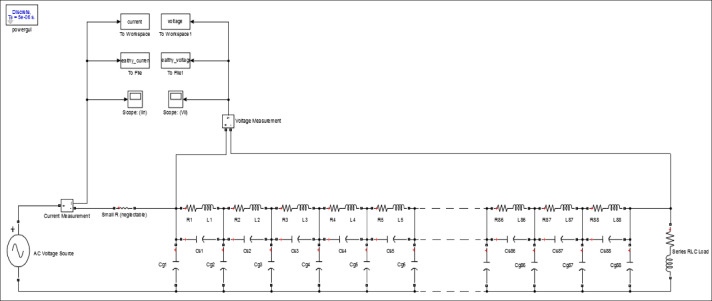
The applied model structures.

An integrated model by applying MATLAB-SIMULINK was implemented for the three transformers models with respect to the pre-mentioned electrical parameters. In the proposed model, an AC voltage supply with a low amplitude and a frequency of 50 Hz is used. The instantaneous values of ΔV and I_in_ are sampled for a duration of 0.02 s (one cycle) with a time step of 10 µsec. The transformer models being tested are constructed for the healthy condition with a load impedance of (8 + j6) Ω. Figure 6 shows the proposed model structure for the transformer under study. The modelling structure keep constant in the case of 3 different transformers model (3, 5 and 7 MVA), only the electrical parameters RLC are changed according to the simulated transformer.

The results from MATLAB-SIMULINK simulations are presented for the three transformers models. Simulation results are classified into three categories: transformer healthy condition (No fault), Transformer healthy condition under harmonics effect (3^rd^, 5^th^ and 7^th^ orders) and transformer abnormal conditions (3 various internal faults) which are explained in detail in the subsequent sections.

### Simulating of healthy condition

**Fig. 7:**
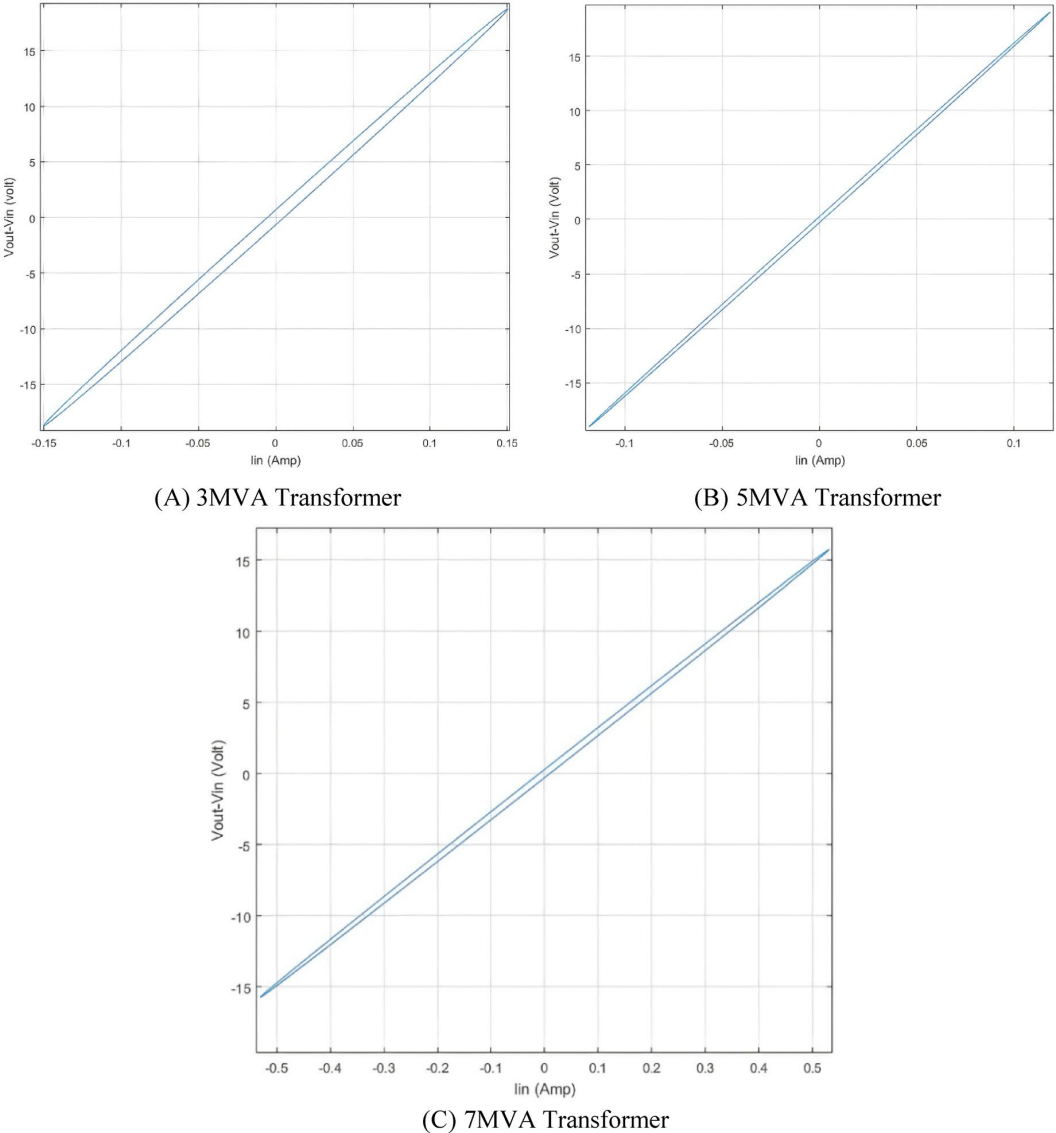
(ΔV—I_in_) locus of 3, 5 and 7 MVA transformers in healthy condition.

The (ΔV—I_in_) locus diagram for three transformer models is generated under healthy conditions with a 0.8 lagging power factor and a load impedance of (8 + j6) Ω. Figure 7 illustrates the healthy condition of the 3, 5, and 7 MVA transformers. Each ellipse represents the unique fingerprint of the corresponding transformer.

From the obtained result in case of 3 different transformer model, for 7 MVA, it is clear that (ΔV–I_in_) locus has the greater value of current (0.5 A) compared with 3 MVA (0.15 A) and 5 MVA (0.14 A). This observation is due to the 7 MVA transformer having the highest value of grounding capacitance 0.12 pF which allows high value of current to pass through the winding. The ground capacitance in 3 and 5 MVA power transformers are 0.08 pF and 0.056 pF respectively.

The narrow ellipse in the three cases is not typical but it almost depends on the physical parameters of winding and the technique of transformer modelling. The way of power transformer parameters modelling directly affects the values of RLC values.

### Simulation of healthy condition under harmonics effect

This section examines the impact of harmonics on the (ΔV—I_in_) locus and presents the harmonic waveform performed on the 3 MVA power transformer model. As illustrated in Table 6, the recommended voltage distortion limits found in IEC 519 at the point of common coupling PPC. These limits should be considered as the system design reference for faulty conditions during normal operation. ^40^

**Table 6:** Limits of voltage distortion according to IEEE standard 519–2014^41^

Bus voltage V at PCC	Individual harmonics (%)	Total harmonic distortion THD (%)
$V\le 1.0 KV$	5.0	8.0
$1.0 KV<V\le 69 KV$	3.0	5.5
$69 KV<V\le 161 KV$	1.5	2.5
$V>161 KV$	1.0	1.5

**Fig. 8:**
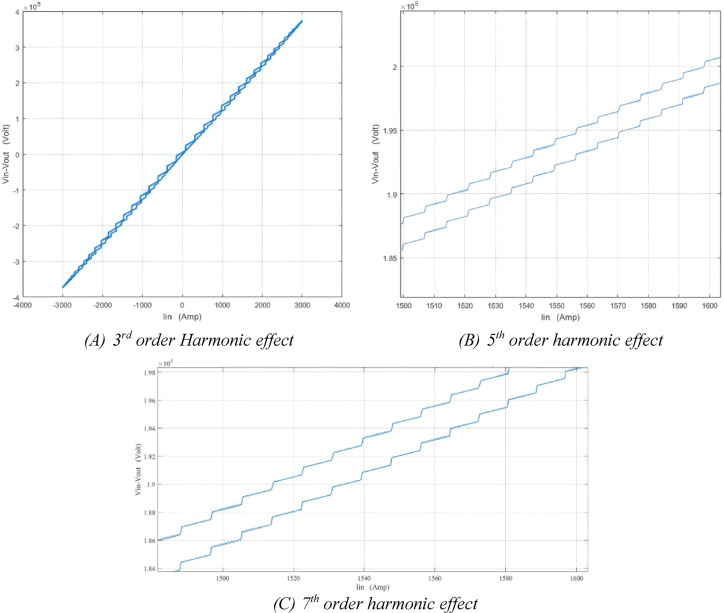
3rd, 5^th^ and 7^th^ orders harmonics effect on (ΔV–I_in_) locus for 3MVA power transformer at healthy condition.

From the data shown in Table 6. It is very clear that the system is run at rated operation 33 kV so, the percentage of individual harmonics distortion set at 3.0%. In this paper 3^rd^ ,5^th^ and 7^th^ harmonics applied on 3 MVA power transformer. Figure 8 shows the effect of harmonics orders on 3 MVA power transformer (ΔV–I_in_) locus.

### Simulating of abnormal conditions

To simulate internal faults within the tested transformers, different insulation failures can be simulated through changing the parameters of the simulated model. For the applied models of 3, 5 and 7 MVA power transformers, three insulation faults are simulated namely turn-to-turn fault, series short circuit and shunt short circuit. The simulation results are compared with the origin fingerprint which is considered the transformer healthy data.

#### Simulation of turn-to-turn fault (TTF)

**Fig. 9:**
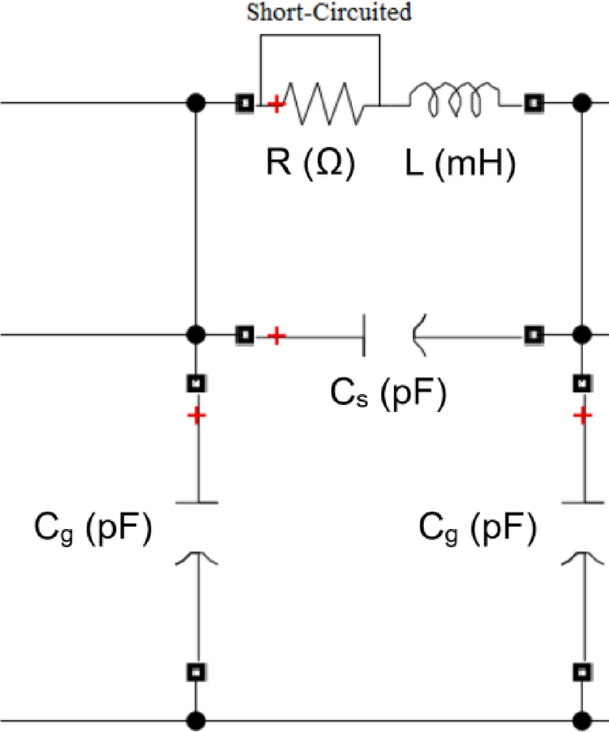
TTF simulation for one disk.

Turn to turn short circuits are responsible for approximately 34% of transformer failures in practice ^12^. In the simulated model, during the TTF simulation, the value of the series resistor must be zero (shorted), as shown in Fig. 9. To examine the impact of TTF on the formulated (ΔV—I_in_) locus, faults at various winding locations are simulated. For the 3 MVA transformer with a total of 89 discs, faults are simulated at 20, 40, 60, and 80 discs. For the 5 MVA transformer, which has 67 total discs, faults are simulated at 15, 30, 45, and 60 faulty discs, and for the 7 MVA transformer with 69 total discs, faults are simulated at 15, 30, 45, and 60 faulty discs, respectively.

**Fig. 10:**
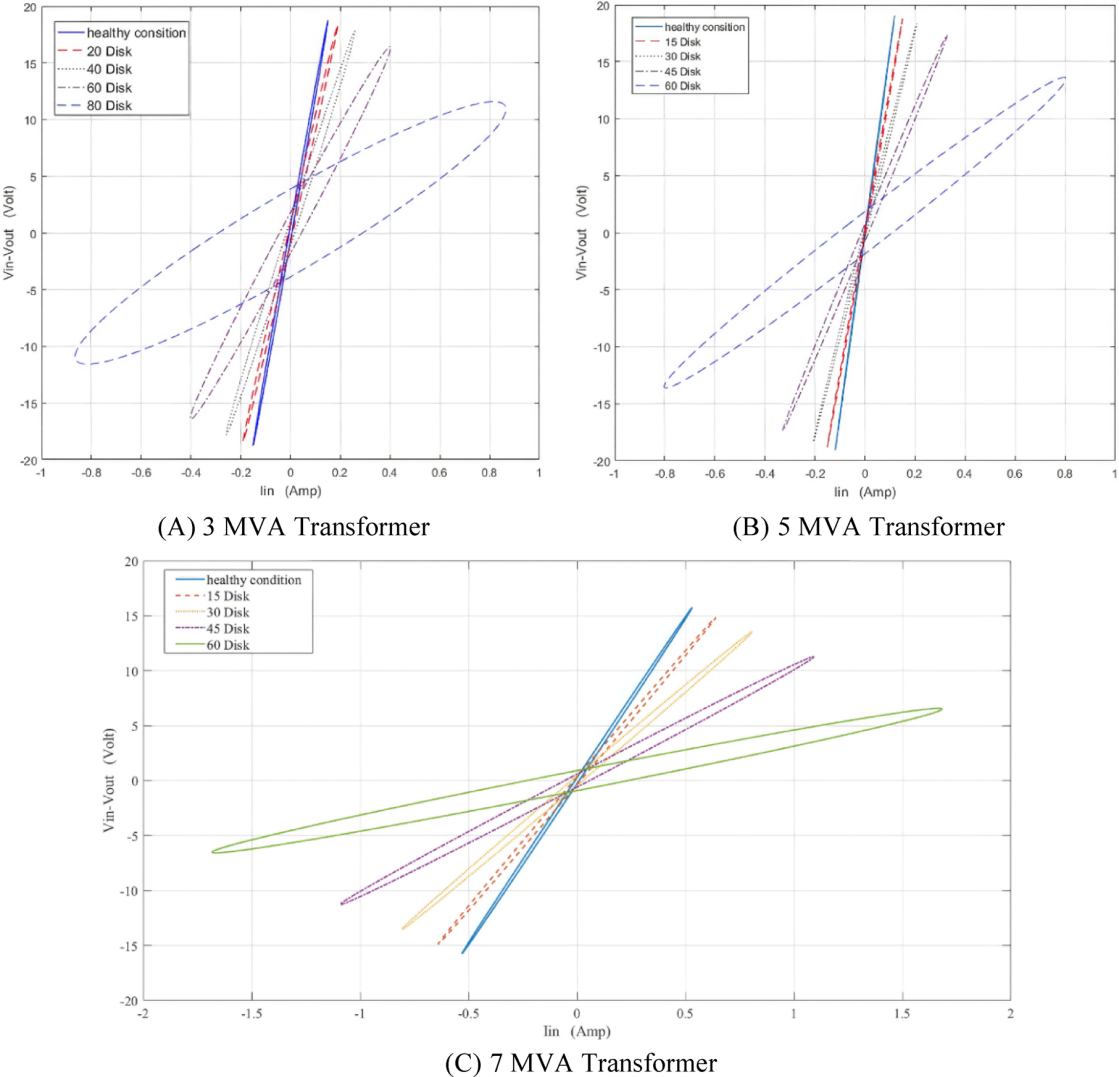
Effect of turn-to-turn fault (TTF) on (ΔV—I_in_) locus for 3,5 and 7 MVA power transformers.

The locus for TTF is displayed in Fig. 10 at different faulty discs for 3, 5 and 7 MVA respectively. The locus rotates clockwise and expands in area as the number of defective discs increases.

#### Simulation of series short circuit (SEF)

**Fig. 11:**
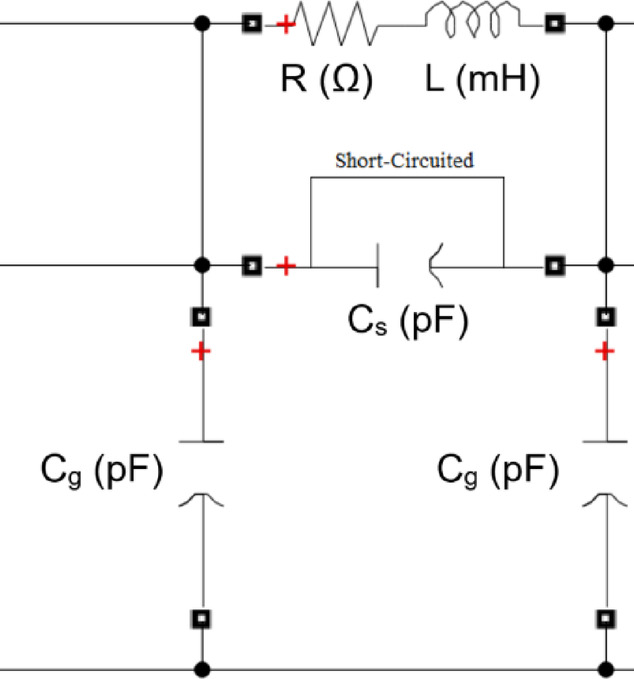
SEF simulation for one disk.

**Fig. 12:**
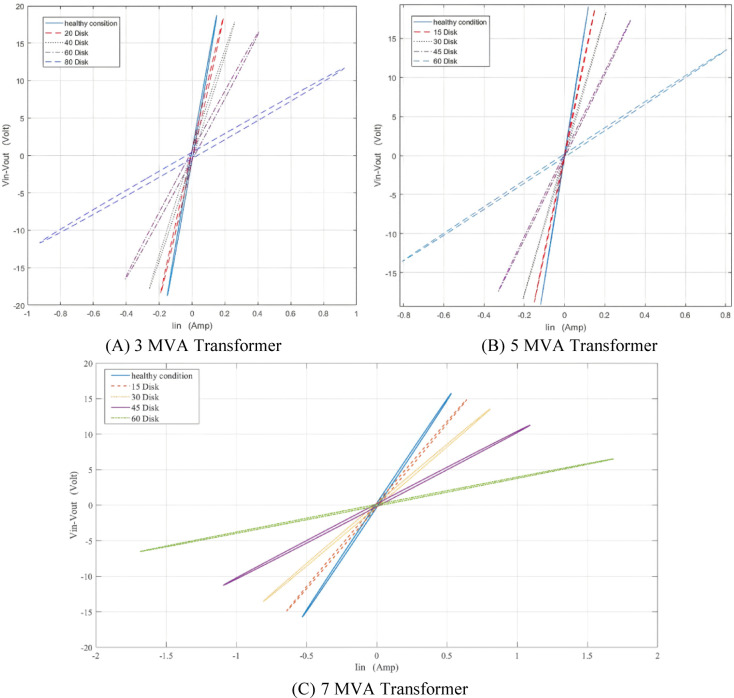
Effect of series short circuit faults (SEF) on (ΔV—I_in_) locus for 3,5 and 7 MVA transformer.

A series fault refers to an insulation breakdown between the discs. In the simulated model, the faulty disc is short-circuited to represent a SEF. Figure 11 shows the representation of SEF at one disk. To evaluate the impact of SEF on the (ΔV–I_in_) locus, the locus is generated for series short-circuit faults occurring at 20, 40, 60, and 80 discs for the 3 MVA transformer, and at 15, 30, 45, and 60 faulty discs for the 5 MVA and 7 MVA transformers. The resulting faulty (ΔV—I_in_) loci are then compared to the healthy condition locus, as shown in Fig. 12 for the 3, 5, and 7 MVA transformers, respectively. It is evident that the locus moves clockwise and gets smaller as the number of defective discs increases.

#### Simulation of shunt short circuit fault (SHF)

**Fig. 13:**
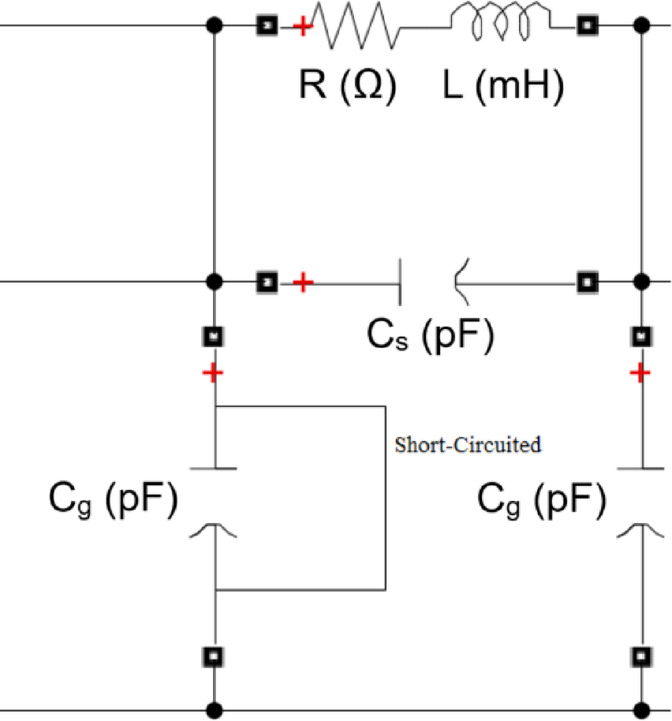
SHF simulation for one disk.

**Fig. 14:**
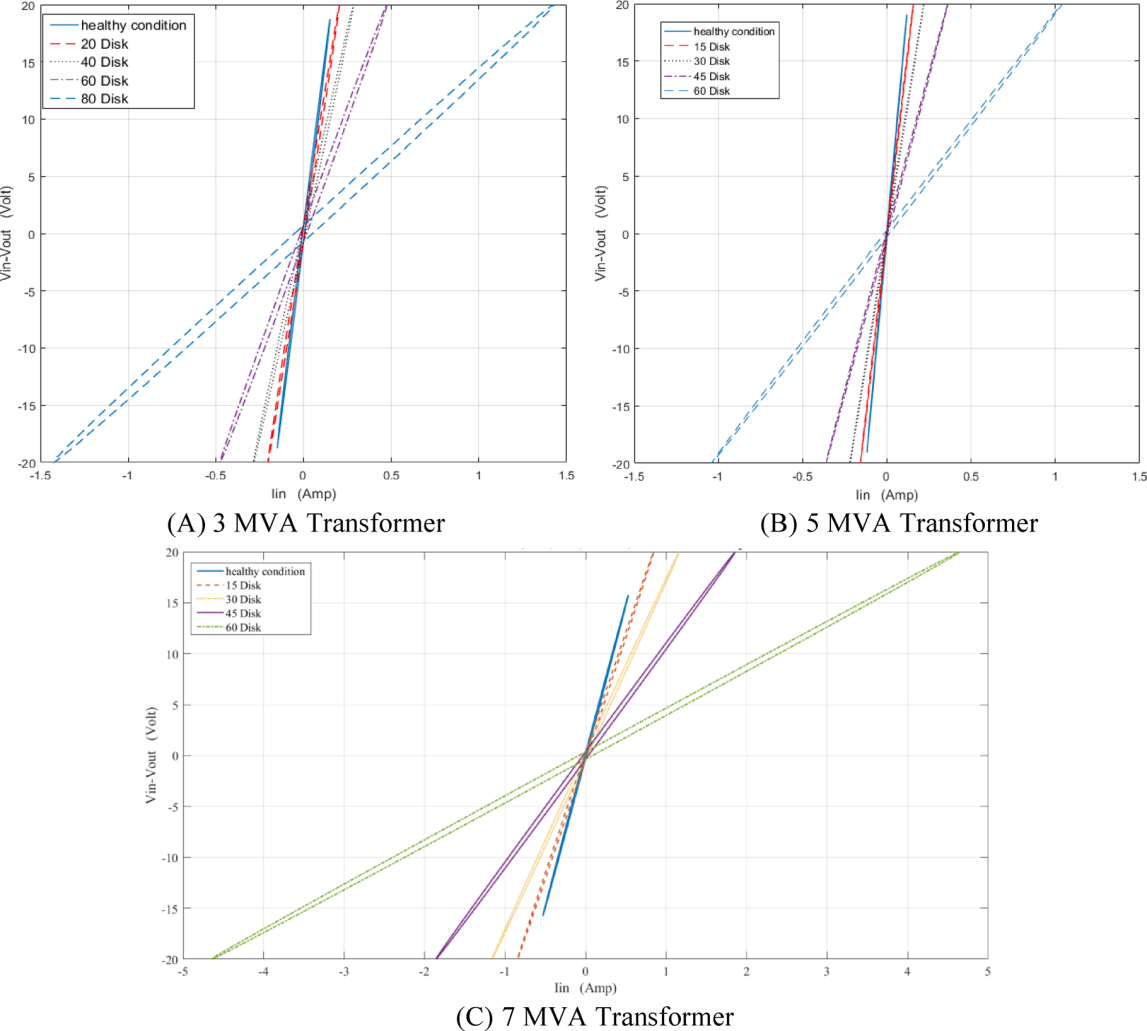
Effect of shunt short circuit faults (SHF) on (ΔV—I_in_) locus for 3,5 and 7 MVA transformer.

Leakage faults or disc to ground faults in a transformer are primarily caused by insulation deterioration, harm to the earth shield, high moisture levels in the windings, hotspots, and insulation aging. Shunt faults, on the other hand, arise from insulation breakdown across the winding and earthed parts such as the core or tank ^37^. In the proposed simulation model, a SHF is represented by connecting the faulty disc to ground, as shown in Fig. 13. Figure 14 illustrates the loci generated for the three transformer models under various faulty disc conditions, compared to the healthy state locus. It is evident that as the number of defective discs increases, the resultant diagram twists clockwise, and its overall area enlarges.

## Features extraction discrimination technique

This section outlines the methodologies employed to distinguish faults within transformer windings, focusing on three distinct types of internal faults applied to three different transformer models. For the technique to be practical and reliable, it must demonstrate high accuracy in fault discrimination. Following the simulation of insulation failures, as presented earlier, the subsequent target of this research is to identify the fault characteristics, including the class and precise location of the fault.

Extracting significant features for internal fault identification within the power transformer is still an open research area. So, some unrivaled features of the ellipse are used to discriminate between various types of insulation failure within power transformer winding. This can be achieved by comparing each of faulty and normal condition loci. Some of significant features are extracted from each of 267 loci (3 fault types $\times$ 89 disks location) in case of 3 MVA power transformer features extraction process. The total features extracted from simulation 5 MVA power transformer winding is 201 loci (3 fault types $\times$ 67 disks location), while to discriminate 7 MVA power transformer internal fault, 207 loci features (3 fault types $\times$ 69 disks location) have been extracted.

**Fig. 15:**
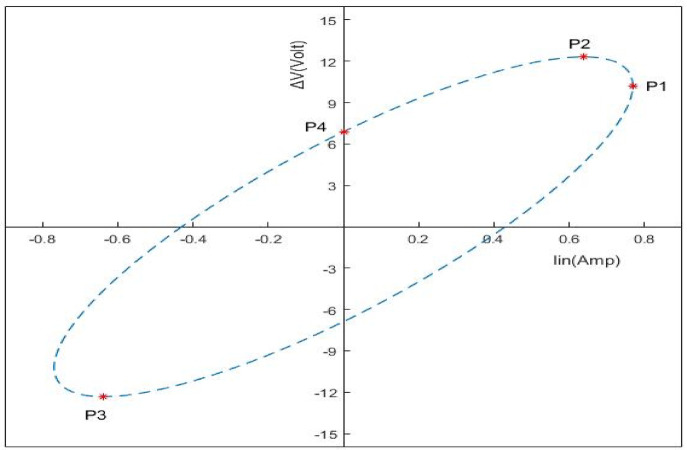
Four essential points on the (ΔV—Iin) locus used to derive the five suggested features.

Five novel features (F1, F2, F3, F4, and F5) were extracted using a MATLAB script designed to analyze the numerical data from the (ΔV–I_in_) locus. These new features which are derived from the developed loci to characterize internal faults. As shown in Fig. 15, The coordinates of four different locations (P1, P2, P3, and P4) are used by the (ΔV–I_in_) locus to calculate the suggested features.

The *x*-coordinate of point P1 determines the first suggested feature, F1, which is equivalent to the greatest value of I_in_. The second and third features, F2 and F3, are based on the y-coordinates of points P2 and P3, which correspond to the minimum and maximum values of ∆V, respectively. The value of F4 is the highest value at point P4, which is the ellipse’s intersection with the y-axis. Finally, the absolute value of the y-coordinate of point P3 is used to compute the fifth feature, or F5.

**Fig. 16:**
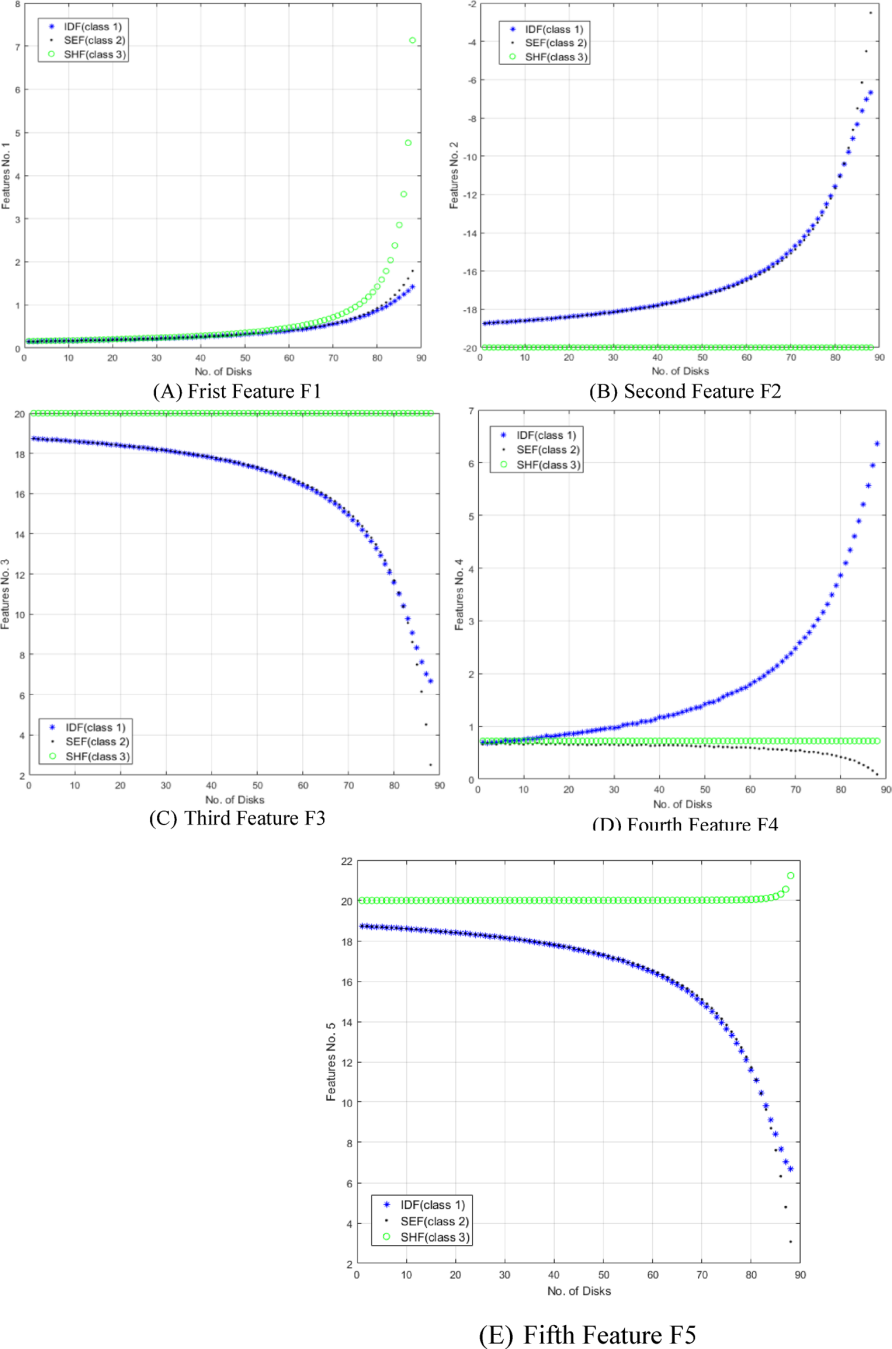
The five features (F1:F5) change against number of defected disks for all fault classes for 3 MVA transformer.

**Fig. 17:**
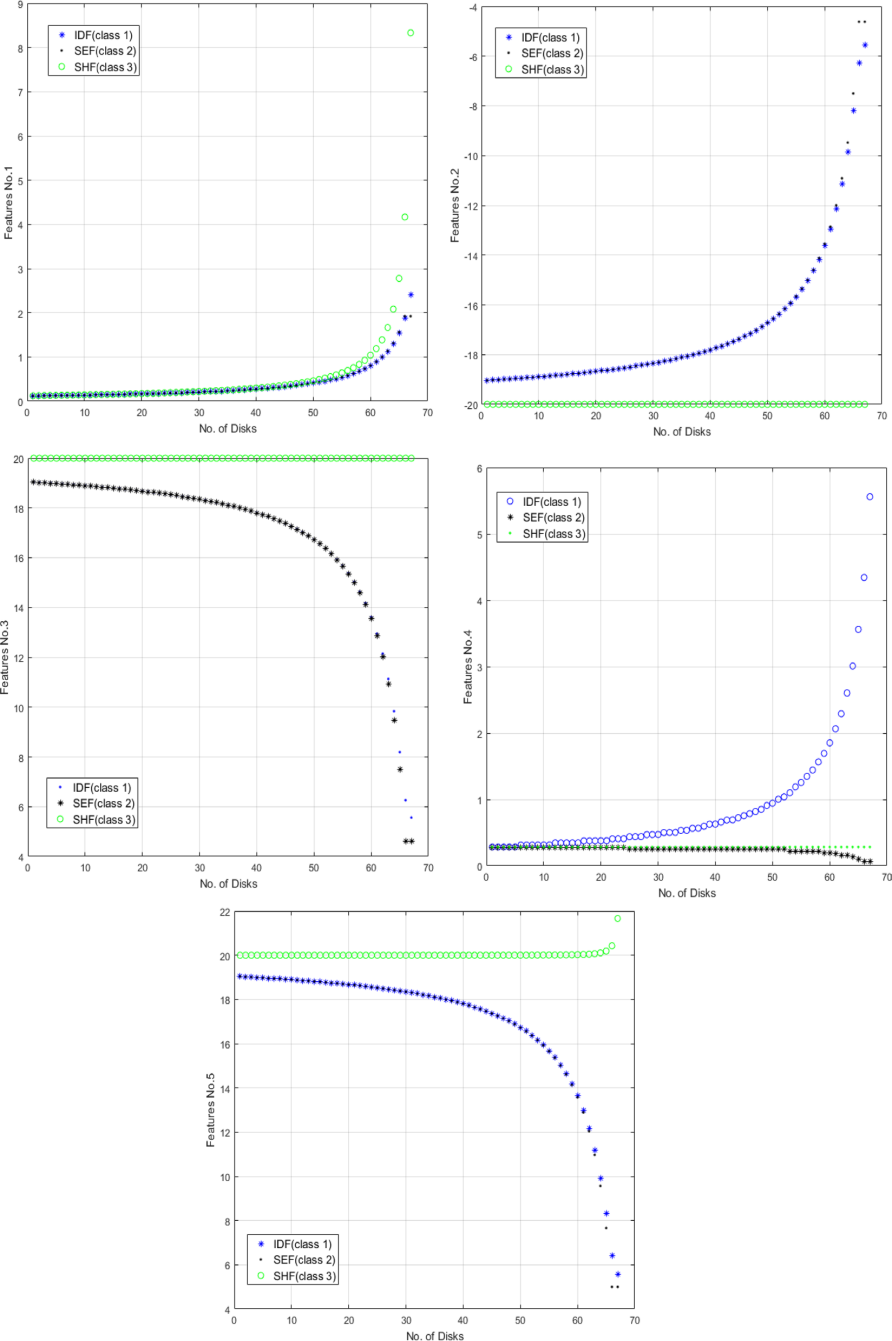
The five features (F1:F5) change against number of defected disks for all fault classes for 5 MVA transformer.

The five proposed features are investigated to effectively discriminate and locate three distinct types of faults. The presented features are extensively analyzed across every fault classification within the transformer to emphasize their effectiveness. Figures 16 and 17 demonstrate the variation of the proposed five features F1, F2, F3, F4 and F5 respectively for all fault types at 89 different fault locations along the transformer winding for 3 MVA power transformer and for 5 MVA power transformer which have 67 total disks respectively.

According to the results obtained in Fig. 16, the five features achieve good discrimination between the various types of internal faults TTF, SEF and SHF. F2, F3 and F5 can separate SHF from other types of faults which leads to high accuracy in shunt short circuit detection. F4 achieves good separation between TTF and SEF which makes the discrimination technique is more accurate and achieves fast detection response. Another developed MATLAB code is applied on 5 MVA power transformer showing the features extracted from the locus. Figure 17 shows the five features (F1:F5) change against number of defected disks for all fault classes for 5 MVA transformer which have 67 total disks. These features can discriminate between TTF, SEF and SHF with high accuracy discrimination for the three types of faults.

For 3 MVA power transformer winding with 89 disks is modeled as 89 nodes. Three internal fault types (TTF, SEF, SHF) are analyzed across these nodes, yielding 267 fault loci (3 fault types × 89 disks). Each locus is characterized by five features, forming a dataset where each fault type has an 89 × 5 matrix (89 disk locations × 5 features). The aggregated input matrix as shown in Eq. 2, combines all fault types into a 267 × 5 structure, while $i$ represent the no. of faulty disks along the transformer winding. the output matrix stated in Eq. 3, labels each row with the fault type and disk location.

2 $$\text{Input Matrix}= \left[\begin{array}{ccccc}{\text{F}1}_{{\text{TTF}}_{1}}& {\text{F}2}_{{\text{TTF}}_{1}}& {\text{F}3}_{{\text{TTF}}_{1}}& {\text{F}4}_{{\text{TTF}}_{1}}& {\text{F}5}_{{\text{TTF}}_{1}}\\ \vdots & \vdots & \vdots & \vdots & \vdots \\ {\text{F}1}_{{\text{TTF}}_{\text{i}}}& {\text{F}2}_{{\text{TTF}}_{\text{i}}}& {\text{F}3}_{{\text{TTF}}_{\text{i}}}& {\text{F}4}_{{\text{TTF}}_{\text{i}}}& {\text{F}5}_{{\text{TTF}}_{\text{i}}}\\ {\text{F}1}_{{\text{SEF}}_{1}}& {\text{F}2}_{{\text{SEF}}_{1}}& {\text{F}3}_{{\text{SEF}}_{1}}& {\text{F}4}_{{\text{SEF}}_{1}}& {\text{F}5}_{{\text{SEF}}_{1}}\\ \vdots & \vdots & \vdots & \vdots & \vdots \\ {\text{F}1}_{{\text{SEF}}_{\text{i}}}& {\text{F}2}_{{\text{SEF}}_{\text{i}}}& {\text{F}3}_{{\text{SEF}}_{\text{i}}}& {\text{F}4}_{{\text{SEF}}_{\text{i}}}& {\text{F}5}_{{\text{SEF}}_{\text{i}}}\\ {\text{F}1}_{{\text{SHF}}_{1}}& {\text{F}2}_{{\text{SHF}}_{1}}& {\text{F}3}_{{\text{SHF}}_{1}}& {\text{F}4}_{{\text{SHF}}_{1}}& {\text{F}5}_{{\text{SHF}}_{1}}\\ \vdots & \vdots & \vdots & \vdots & \vdots \\ {\text{F}1}_{{\text{SHF}}_{\text{i}}}& {\text{F}2}_{{\text{SHF}}_{\text{i}}}& {\text{F}3}_{{\text{SHF}}_{\text{i}}}& {\text{F}4}_{{\text{SHF}}_{\text{i}}}& {\text{F}5}_{{\text{SHF}}_{\text{i}}}\end{array}\right]$$

3 $$\text{Output Matrix}=\left[\begin{array}{c}{\text{TTF}}_{1}\\ \vdots \\ {\text{TTF}}_{\text{i}}\\ {\text{SEF}}_{1}\\ \vdots \\ {\text{SEF}}_{\text{i}}\\ {\text{SHF}}_{1}\\ \vdots \\ {\text{SHF}}_{\text{i}}\end{array}\right]$$

## Internal fault discrimination results

In this work, Artificial Neural Networks (ANNs) with Learning Vector Quantization (LVQ) were intentionally selected over more complex deep learning architectures such as CNNs or LSTMs due to the nature and dimensionality of the dataset. The proposed method extracts five meaningful features from the ΔV-I_in_ locus, resulting in a compact dataset (267 samples × 4 features) that is well-suited for lightweight, fast-converging models like LVQ-based ANNs. Moreover, deep learning models typically require large-scale, high-dimensional data and significantly more computational resources, which may not be justified for this specific application.

**Fig. 18:**
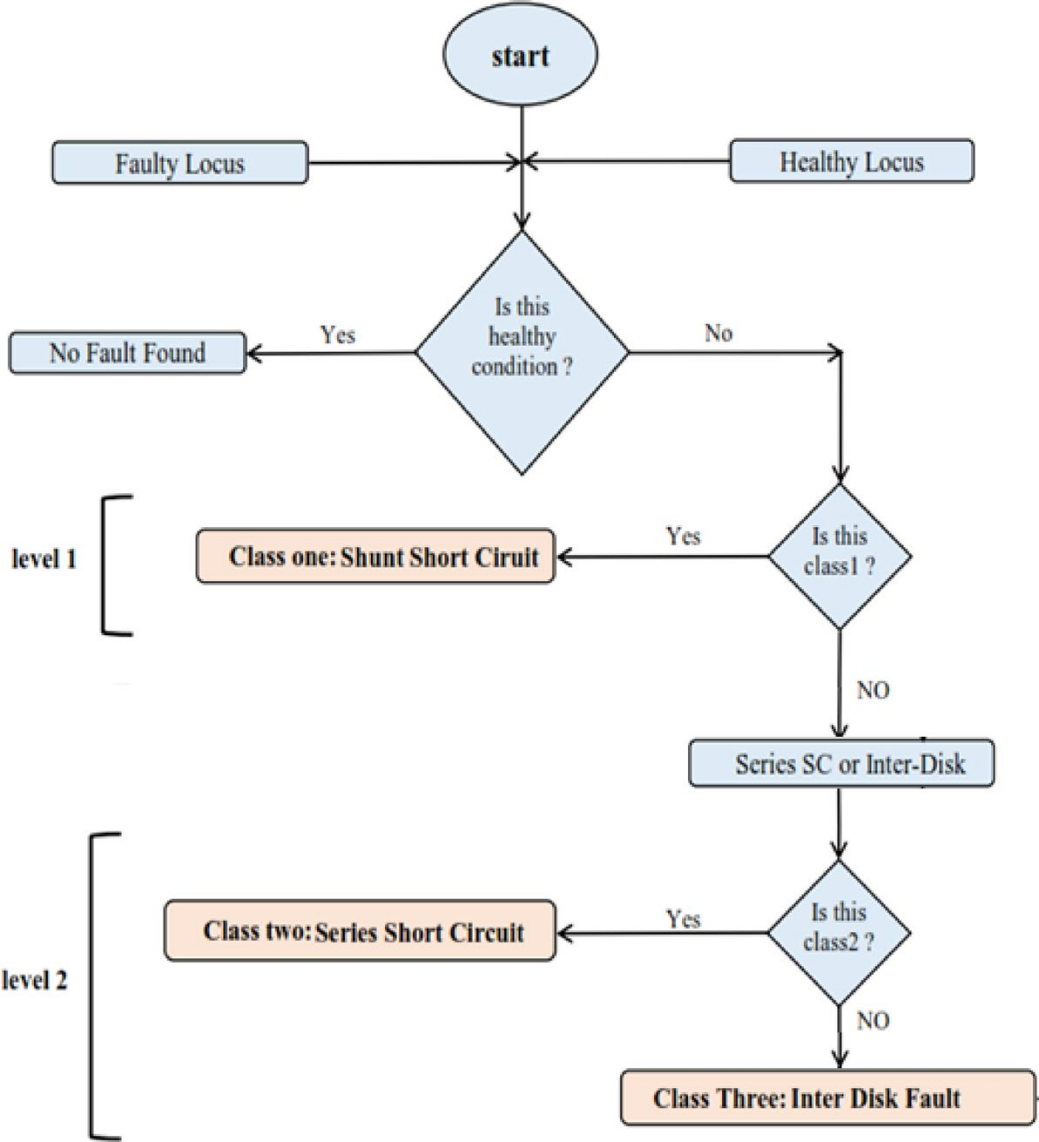
General flowchart that describes the fault classification.

A multi-class LVQ classifier processes extracted features from faulty ΔV-I_in_ loci to identify insulation failure types. The results of fault identification using LVQ indicate that the selected number of features is sufficient to achieve reasonable accuracy in the classification task. To enable ANNs to perform classification, a large dataset of faulty condition records is used to train the network. During the process, it was observed that the data matrix for fault identification could be reduced to 267 rows and 4 columns (selecting four out of the five features). This minimization helps streamline the ANN training procedure while maintaining sufficient achievement. Figure 18 illustrates the general logic flowchart for the fault classification process, which is implemented using a two-level LVQ algorithm comprising ANN 1 and ANN 2, as follows:

- *Level 1 (ANN 1)*: At the first level, the LVQ model (ANN 1) focuses on identifying shunt short circuit faults. However, it is unable to differentiate between the other two fault types—series short circuits and turn-to-turn fault—at this stage.
- *Level 2 (ANN 2)*: To address this limitation, the second level of the LVQ model (ANN 2) is utilized. This level is specifically designed to classify the remaining faults, effectively distinguishing between turn-to-turn faults and series short circuits within the dataset.
- A two-level neural network is used to discriminate between the three types of faults because there is a large similarity between TTF and SEF.
- The proposed LVQ-ANN comprises five subnetworks (ANN1–ANN5), each tailored for specific fault identification and localization tasks. The parameter configurations for these networks, including the number of neurons, learning rates, and layer designs, are summarized in Table 7. ANN1 and ANN2 focus on fault identification, while ANN3–ANN5 are dedicated to fault localization (detailed in Sect. “Internal fault location results”).

**Table 7:** Parameter configurations for LVQ-ANN components (ANN1–ANN5).

Network	Task	No. of Neurons	Learning Rate	Training/Testing Split	No. of Layers	Activation Functions
ANN1	Identify SHF	3	0.1	50%: 50% (132/135) cases	1	ReLU (hidden), Sigmoid (output)
ANN2	Classify SEF, TTF	7	0.07		1	ReLU (hidden), Sigmoid (output)
ANN3	Locate SHF	12	0.05		3	ReLU (hidden), Linear (output)
ANN4	Locate TTF	4	0.08		1	SoftMax (output)
ANN5	Locate SEF	8	0.06		2	Tanh (hidden), SoftMax (output)

To ensure reproducibility and reduce the non-deterministic nature of ANN training, a fixed random seed was applied during all training sessions. Each ANN subnetwork was trained across 10 independent runs, and the best-performing model—based on highest validation accuracy—was selected. The training/testing dataset split was maintained consistently at 50/50. All network architectures and parameters were kept constant as detailed in Table 7.

As shown in Table7, The LVQ subnetworks employ activation functions to introduce nonlinear decision boundaries. Hidden layers use Rectified Linear Units (ReLU), while output layers utilize Sigmoid (binary tasks) or SoftMax (multi-class tasks). The LVQ prototypes are updated using Euclidean distance and a decaying learning rate.

To rigorously assess the proposed LVQ algorithm, three complementary metrics: Precision, Recall, and F1-Score, were selected. While accuracy provides a surface-level measure of overall correctness, it fails to address critical nuances such as fault types. Precision quantifies the reliability of positive predictions as shown in Eq. (4). Recall evaluates the model’s ability to capture all relevant cases as stated in Eq. (5), and the F1-Score harmonizes these metrics into a single robust indicator as displayed in Eq. (6). A confusion matrix was further employed to visualize the model’s performance across fault classes, offering granular insights into true positives (TP), false positives (FP), and false negatives (FN). This matrix not only clarifies the distribution of correct and incorrect predictions but also highlights patterns in misclassification (distinguishing Between TTF, SEF and SHF). By synthesizing these metrics and visual tools, the evaluation framework ensures a rigorous, interpretable, and bias-resistant validation of the LVQ algorithm’s diagnostic capabilities.

4 $$\text{Precision}= \frac{{T}_{P}}{{T}_{P}+{F}_{P}}$$

5 $$\text{Recall}= \frac{{T}_{P}}{{T}_{P}+{F}_{N}}$$

6 $$F1-\text{Score}= \frac{2*\text{Precision}*\text{Recall}}{\text{Precision}+\text{Recall}}$$

**Fig. 19:**
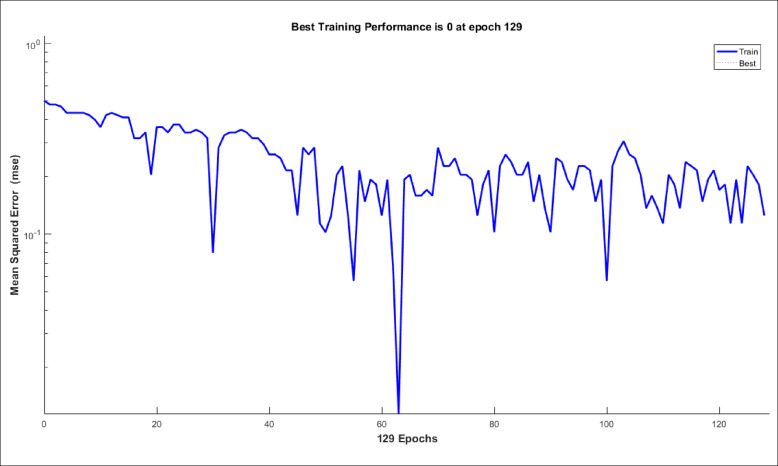
Training performance of the LVQ-ANN model.

The proposed LVQ-ANN model’s training performance is illustrated in Fig. 19, which plots the Mean Squared Error (mse) against the number of training epochs. The model achieved perfect convergence (MSE = 0) at epoch 129, with the error decreasing rapidly from an initial value of 120. Intermediate MSE values at epochs 5, 10, 20, 40, 60, 80, 100, and 120 reflect progressive refinement of prototype vectors. The sharp decline in MSE during early epochs indicates efficient feature learning, while the gradual stabilization toward zero suggests careful tuning of prototypes to avoid overfitting. This aligns with the high testing accuracy (98.51%) achieved in fault identification, confirming robust generalization despite perfect training performance.

According to achieved results, it is found that the identification scheme has properly identified 265 fault loci from 267 total faulty loci. This process is achieved after 42 s training time using 4 features through using 2-levels LVQ network. As presented in Table 8, this identification scheme achieves an overall accuracy of approximately 98.51%. Table 9 summarize the detailed distribution of correct and misclassified predictions.

**Table 8:** Overall fault identification results.

Metric	Turn-To-turn fault	Series short circuit	Shunt short circuit
Tested cases	45	45	45
True positives (TP)	43	45	45
False positives (FP)	0	0	0
False negatives (FN)	2	0	0
Precision	100%	100%	100%
Recall	95.55%	100%	100%
F1-Score	97.7%	100%	100%
Overall Accuracy	98.51%

**Table 9:** Confusion matric for fault identification approach.

Predicted actual	Turn-to-turn fault	Series short circuit	Shunt short circuit
Turn-to-Turn Fault	43	0	2
Turn-to-Turn Fault	0	45	0
Turn-to-Turn Fault	0	0	45

## Internal fault location results

A LVQ multi-level algorithm for sub-classification is proposed to identify the locations of predefined fault types. In this approach, the high-voltage winding of the transformer is split into four segments. Sect. “Introduction” includes disks numbered from 1 to 22. Section “Paper contribution” continues from disk numbers 23 to 44. Section “Transformer model contains internal faults” follows, encompassing disks 45 to 66, while Sect. “Proposed method” concludes the segmentation with disks numbered from 67 to 89.

The sub-classification LVQ algorithm is implemented for finding the location of each fault after ending the time simulated of the previous 2-levels LVQ algorithm which belongs to fault identification process.

In this stage, the data matrix that is used for the multi-level LVQ localization algorithm includes 267 positions records (rows) and 5 features (columns). The fault location procedure is carried out Appling five separate ANNs (ANN 3 through ANN 5), with each ANN dedicated to a particular defect class. The roles and descriptions of these five ANNs are outlined as follows:

- *ANN 3*: This network, structured in three levels, is responsible for locating the faulty section resulting from a shunt short circuit. The three-level design is implemented to account for the similarity in features within each faulty section.
- *ANN 4*: In this stage, the location of turn-to-turn fault can be found through 1-levels LVQ network.
- *ANN 5*: this level can distinguish the number of faulty disks for series short circuit fault inside power transformer winding.
- The LVQ-ANN subnetworks (ANN3–ANN5) utilize the parameter configurations detailed in Table 7 to localize faults across transformer winding segments.

In the insulation failure location process, key features are extracted from each data point. The algorithm accurately located 258 out of 267 faulty disks in 1 min and 52 s of training time, using 5 features across 3 LVQ networks. Each fault type is localized using a dedicated LVQ network with 4 critical features. The fault location accuracy of TTF, SEF and SHF has been achieved as illustrated in Table 10, Table 11 and Table 12 respectively, with confusion matrices highlighting correct and misclassified sections for TTF, SEF and SHF displayed in Table 13, Table 14 and Table 15 respectively. The total fault location precision across all fault types is 93.275, as summarized in Table 16.

**Table 10:** turn-to-turn fault location results.

Section	Tested cases	True positives (TP)	False positives (FP)	False negatives (FN)	Precision	Recall	F1-Score
Section 1	11	11	0	0	100%	100%	100%
Section 2	11	10	1 (predicted Sec 3)	1	90.9%	90.9%	90.9%
Section 3	11	10	1 (predicted Sec 2)	1	90.9%	90.9%	90.9%
Section 4	12	11	1 (predicted Sec 3)	1	91.67%	91.67%	91.67%
Overall result	45	42	3	3	93.36%	93.36%	93.36%

**Table 11:** Series short circuit fault location results.

Section	Tested cases	True positives (TP)	False positives (FP)	False negatives (FN)	Precision	Recall	F1-Score
Section 1	11	11	0	0	100%	100%	100%
Section 2	11	10	1 (predicted Sec 3)	1	90.9%	90.9%	90.9%
Section 3	11	10	1 (predicted Sec 4)	1	90.9%	90.9%	90.9%
Section 4	12	12	0	0	100%	100%	100%
Overall result	45	43	2	2	95.45%	95.45%	95.45%

**Table 12:** Shunt short circuit fault location results.

Section	Tested cases	True positives (TP)	False positives (FP)	False negatives (FN)	Precision	Recall	F1-Score
Section 1	11	10	1 (predicted Sec 2)	1	90.9%	90.9%	90.9%
Section 2	11	10	1 (predicted Sec 1)	1	90.9%	90.9%	90.9%
Section 3	11	10	1 (predicted Sec 4)	1	90.9%	90.9%	90.9%
Section 4	12	11	1 (predicted Sec 3)	1	91.67%	91.67%	91.67%
Overall result	45	41	4	4	91.0175%	91.0175%	91.0175%

**Table 13:** Confusion matrix for TTF localization approach.

Predicted actual	Section 1	Section 2	Section 3	Section 4
Section 1	11	0	0	0
Section 2	0	10	1	0
Section 3	0	1	10	0
Section 4	0	0	1	11

**Table 14:** Confusion matrix for SEF localization approach.

Predicted actual	Section 1	Section 2	Section 3	Section 4
Section 1	11	0	0	0
Section 2	0	10	1	0
Section 3	0	0	10	1
Section 4	0	0	0	12

**Table 15:** Confusion matrix for SHF localization approach.

Predicted actual	Section 1	Section 2	Section 3	Section 4
Section 1	10	1	0	0
Section 2	1	10	0	0
Section 3	0	0	10	1
Section 4	0	0	1	11

**Table 16:** Overall fault location results.

Fault type	Fault location accuracy				
	Section 1	Section 2	Section 3	Section 4	Overall accuracy
Turn-To-Turn Fault	100%	90.9%	90.9%	91.67%	93.36%
Series Short Circuit	100%	90.9%	90.9%	100%	95.45%
Shunt Short Circuit	90.9%	90.9%	90.9%	91.67%	91.0175%
Overall Fault Location Accuracy	93.275%				

## Comparison of the proposed scheme with other methods

Table 17 provides a concise comparison between the proposed method and previously published approaches for fault identification and location.

**Table 17:** Comparison of previous and proposed method for fault detection and location.

References	Fault detection method	Classification technique	Noise consideration	Complexity	Data quantity	Threshold dependence	Detection time	Training/testing ratio	Fault identification accuracy	Fault location accuracy
^42^	Random Forest (RF)	Ensemble decision trees	Yes	Moderate	3240 Cases	No	1 cycle	50% to 50%	98.33%	N/R
^43^	XGBoost	CNN	No	High	141,768 cases	No	1 cycle (22 ms)	63% to 37%	99.95%	N/R
^44^	Dissolved Gas Analysis (DGA)	SVM optimized by Genetic Algorithm (GA)	No	Moderate	235 cases	No	N/R	fivefold cross-validation	87.18%	N/R
^45^	Cross Correlation Features	Multi-layer Perceptron (MLP) Artificial Neural Network (ANN)	No	Moderate	86 cases	No	N/R	70% to 30%	97.9%	80.3%
^46^	Transfer function measurement	probabilistic neural network (PNN)	No	High	107 cases	No	N/R	75% to 25%	94.7%	N/R
^47^	Transfer function analysis	Support Vector Machine (SVM)	No	High	116 cases	No	N/R	83.62% to 16.38%	94.7%	N/R
^48^	DGA (Dissolved Gas Analysis)	Self-organizing neural network with incremental learning, combined with k-means clustering	No	Reduced computational complexity	849 cases	Yes	N/R	70% to 30%	93.7%	N/R
^49^	Frequency Response Analysis (FRA)	Probabilistic Neural Network (PNN), Decision Tree (DT), Support Vector Machine (SVM), k-Nearest Neighbors (k-NN)	No	High	36 cases	No	N/R	fivefold cross-validation 80% to 20%	100% for SVM	N/R
^50^	Hybrid Wavelet-CNN	Fully Convolutional Network (FCN), Multilayer Perceptron (MLP)	Yes	Moderate	N/R	Yes	N/R	N/R	97% (FCN Model), 75% (MLP Model)	N/R
^51^	FRA with Convolutional Neural Network (CNN)	Graph CNN, CNN, MLP and SVM	Yes	Low	272 cases	No	N/R	70% to 30%	98.33%	N/R
Proposed method	(ΔV-I_in_) locus	Learning Vector Quantization (LVQ)	No	moderate	675 cases	No	1 cycle	50% to 50%	98.51%	93.275%

## Conclusion

This paper investigates three common transformer winding faults—turn-to-turn fault, series short-circuit, and shunt short-circuit—at various locations by simulating power transformers with different ratings. These simulations include a 33 kV winding for 3 MVA and 5 MVA transformers, as well as a 20 kV winding for a 7 MVA transformer. The proposed online scheme effectively distinguishes and identifies the locations of these insulation failures. The scheme utilizes input voltage, output voltage, and input current data to generate the (ΔV–I_in_) locus. Minor variations in the (ΔV–I_in_) locus, caused by different types of insulation failures, are detected in real-time using specific features.

The (ΔV–I_in_) locus serves as an effective tool for monitoring changes in the transformer. In this research, the effects of various harmonic orders—namely the 3^rd^, 5^th^, and 7^th^ harmonics—are examined when applied to a 3 MVA power transformer. The results demonstrate how these harmonic orders influence the (ΔV–I_in_) locus of the transformer. To improve the accuracy of insulation fault identification, new features representing fault characteristics are extracted from the developed ellipse. These features are derived from the (ΔV–I_in_) locus for 3, 5, and 7 MVA power transformers under various fault conditions.

A multi-class neural network algorithm was developed using training sets derived from extracted features to detect deformations in power transformers. The system incorporates two LVQ levels, due to the similarities between certain fault features. The results of the fault identification, based on LVQ, demonstrate that four features are sufficient to achieve a high level of accuracy. The proposed algorithm successfully identified 265 faulty loci out of 267 in just 42 s using the extracted features. As a result, the algorithm achieved an identification accuracy of approximately 98.51%.

A multi-level neural network approach is introduced for identifying insulation failure locations within the winding disks of power transformers, utilizing the same analytical features. The high-voltage winding of the transformer is segmented into four segments. The fault detection method employs three levels of Learning Vector Quantization (LVQ) to accurately pinpoint the specific disk experiencing failure within the transformer winding. The algorithm demonstrated its effectiveness by correctly locating 258 out of 267 faulty disks, achieving a training duration of 1 min and 52 s. As a result, the proposed method attained an overall localization accuracy of approximately 93.275%.

## Data Availability

The datasets generated and/or analyzed during the current study are not publicly available due to the proprietary nature of the measurement setup and data processing techniques. However, the data are available from the corresponding author upon reasonable request.
